# Interfacial Reactions and Mechanical Properties Studies of C-Coated and C/B_4_C Duplex-Coated SiC Fiber-Reinforced Ti_2_AlNb Composites

**DOI:** 10.3390/ma12193257

**Published:** 2019-10-06

**Authors:** Shuming Zhang, Minjuan Wang, Mao Wen, Jianhong Chen, Hu Li, Chuan Xie, Wangtengfei Fan, Qingfeng Wang, Hao Huang

**Affiliations:** 1 State Key Laboratory of Metastable Materials Science and Technology, Yanshan University, Qinhuangdao 066004, China; smzhang89@yeah.net; 2 AECC Beijing Institute of Aeronautical Materials, P.O.Box: 81-15, Beijing 100095, China; 3 State Key Laboratory of Superhard Materials, Department of Materials Science, Key Laboratory of Automobile Materials, MOE, Jilin University, Changchun 130012, China

**Keywords:** SiC_f_/Ti_2_AlNb composite, C coating, C/B_4_C duplex coating, interfacial reaction, tensile strength

## Abstract

Continuous SiC fiber-reinforced Ti_2_AlNb matrix composites have a great potential for high-temperature aviation structure applications, and their properties strongly depend on the microstructure of the interfacial reaction layer. Notably, introducing diffusion barrier coatings has still been a popular strategy for optimizing the interfacial structure and interfacial properties of SiC_f_/Ti. In this work, C coating and C/B_4_C duplex coating were successfully fabricated onto SiC fibers via chemical vapor deposition (CVD), then consolidated into the SiC_f_/C/Ti_2_AlNb and the SiC_f_/C/B_4_C/Ti_2_AlNb composites, respectively, via hot isostatic pressing (HIP) under the condition of 970 °C, 150 MPa, 120 min, and finally furnace cooled to room temperature. The C- and C/B_4_C-dominated interfacial reactions in the SiC_f_/C/Ti_2_AlNb and the SiC_f_/C/B_4_C/Ti_2_AlNb were explored, revealing two different reaction products sequences: The different-sized TiC and the coarse-grained (Ti,Nb)C + AlNb_3_ for the SiC_f_/C/Ti_2_AlNb; and the fine-grained TiB_2_ + TiC, the needle-shaped (Ti,Nb)B_2_/NbB + (Ti,Nb)C, the coarse-grained (Ti,Nb)C + AlNb_2_ for the SiC_f_/C/B_4_C/Ti_2_AlNb. Annealing experiments were further carried out to verify the different reaction kinetics caused by C coating and C/B_4_C duplex coating. The reaction layer (RL)-dominated interfacial strength and tensile strength estimations showed that higher interface strength and tensile strength occurred in the SiC_f_/C/Ti_2_AlNb instead of the SiC_f_/C/B_4_C/Ti_2_AlNb, when the same failure mode of fiber push-out took place.

## 1. Introduction

**Figure 1:**
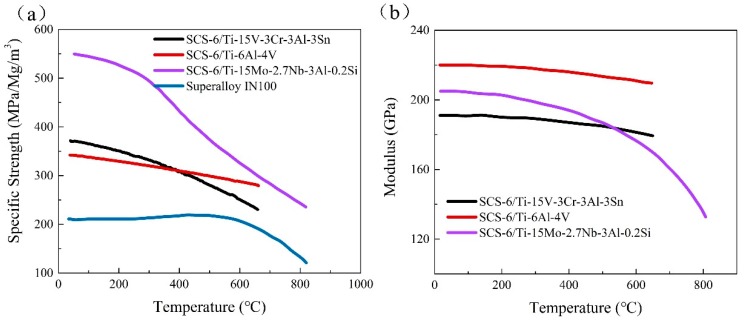
Specific strength (**a**) and modulus (**b**) of several unidirectional TMCs and a nickel-based superalloy.

Continuous SiC fiber-reinforced titanium matrix composites (SiC_f_/Ti) have been considered as one of most potential light-weight high-strength structural materials required by aerospace vehicles and advanced propulsion system, due to their high specific strength and stiffness at both room and elevated temperature [1,2,3]. The physical and mechanical properties of several materials for potential applications in the aero-engine field are shown in Table 1 [4]. And the specific strength and stiffness at room and elevated temperatures of several composites are shown in Figure 1 [5]. Raising the service temperature of SiC_f_/Ti is a continuous pursuit driven by many new applications. Recently, intermetallic titanium aluminides (TiAl, Ti_3_Al, Ti_2_AlNb) have begun to be employed as the matrix of SiC_f_/Ti due to their more excellent high temperature performance, as compared with common Ti alloys [6,7]. Among them, Ti_2_AlNb alloys with higher ductility and formability have attracted more attention due to their excellent low- and high-temperature loading capabilities [7,8,9,10]. Therefore, Ti_2_AlNb alloys, especially with O-phase as the main component that contributes a superior ductility [10], would be the potential choice for the development of high-temperature titanium matrix composites (TMCs) [11,12]. Smith et al. fabricated the SCS-6 SiC_f_/Ti_2_AlNb by foil–fiber–foil processing (FFF) and identified the interfacial products consisting of TiC_1-x_+Ti_5_Si_3_, (Ti,Nb,Si)C_1–x_, Al(Ti,Nb)_3_C and (Ti,Nb,Al)_5_(Si,Al)_3_, with the primary reaction zone size of ~0.6 μm; the SiC_f_/Ti_2_AlNb exhibited more excellent high temperature performance compared to SiC_f_/α_2_-Ti and the longitudinal tensile properties of the composite (which are dominated by the fiber strength) were slightly improved due to the orthorhombic matrix. [13]. Luo et al. further introduced C/Mo duplex coatings as barrier layers and prepared the SiC_f_/C/Mo/Ti_2_AlNb by the FFF method. They found that the Mo barrier layer can regulate the microstructure of the matrix by forming a B_2_-riched transition zone between the reaction layer (RL) and the matrix in the SiC_f_/Ti-21Al-29Nb composite, obtaining a higher tensile strength relative to the Ti_2_AlNb alloy [14]. In addition, Yang et al. employed a matrix-coated fiber (MCF) method to fabricate SCS-6 SiC_f_/Ti_2_AlNb, and also explored the interfacial reactions between SCS-6 SiC fiber and Ti_2_AlNb, showing a milder interfacial reaction than that in SiC_f_/super α_2_-Ti (Ti-25Al-10Nb-3V-1Mo) [15]. Compared with other Ti alloys matrices (Ti-6Al-4V, TC17, IMI834) [5,16,17], Ti_2_AlNb acting as the matrix can exactly elevate the service temperature of SiC_f_/Ti. However, the SiC_f_/Ti_2_AlNb is still inadequately understood, especially for the reason that the interfacial reactions of SiC_f_/Ti_2_AlNb are sensitive to the type of diffusion barrier coating, the microstructure of the matrix, the consolidation process, etc.

It is accepted that the interfacial reactions (mainly including interfacial products and their distribution, and each sub-layer thickness) of SiC_f_/Ti would determine the interfacial properties, and further act on the performances of SiC_f_/Ti [2,18]. Accordingly, understanding and further tailoring the interfacial behaviors of SiC_f_/Ti_2_AlNb would be necessary to ensure its superior properties. Notably, introducing diffusion barrier coatings is still a popular strategy for optimizing the interfacial structure and interfacial properties of SiC_f_/Ti, which can not only protect the SiC fiber suffering from the attack from the matrix, but also obtain a moderate interfacial debonding strength arising from the formation of an appropriate interfacial reaction layer. Several potential diffusion barrier coatings, including carbon, carbides, borides, oxides, nitrides, and transition metals, have been investigated to protect SiC fiber and control the interfacial reactions of SiC_f_/Ti [19,20,21,22,23]. Among them, C coating is still the most widely used diffusion barrier coatings in the SiC_f_/Ti [24,25]. In addition to protecting the SiC fiber, the C coating has the following advantages: (1) It has a good thermal expansion coefficient with SiC fiber; (2) it has a good compatibility with SiC; (3) the interface reaction of composites is easy to control; and (4) the process stability is good. The interfacial reactions between SCS-6 SiC fiber and Ti_2_AlNb have been well explored, when the SCS-6 SiC fiber is comprised of surface C coating with some SiC particles in it [15]. However, the interfacial reactions between pure C coating and Ti_2_AlNb matrix are still unclear. In addition, Zhang et al. found that as B_4_C coating was introduced to SiC_f_/TiAl as the diffusion barrier coating, a thin interfacial layer appeared, confirming the role of B_4_C coating in serving as a potential barrier coating in the SiC_f_/TiAl [26]. Obviously, both C and B_4_C are two potential diffusion barrier coatings in the SiC_f_/Ti_2_AlNb. A comparative study on the interfacial reactions of SiC_f_/Ti_2_AlNb with C and B_4_C diffusion barrier coatings would; thus, be helpful to understand their interfacial behaviors.

In the present paper, C coating and C/B_4_C duplex coatings were, respectively, prepared on SiC fibers by chemical vapor deposition (CVD) as different diffusion barrier layers, Then two kinds of SiC_f_/C/Ti_2_AlNb and SiC_f_/C/B_4_C/Ti_2_AlNb composites were successfully fabricated by MCF method and hot isostatic pressing (HIP) consolidation. Finally, C and C/B_4_C diffusion barrier layer-dominated interfacial reactions, matrix microstructure evolutions, interfacial debonding strength, and tensile strength were investigated.

## 2. Materials and Methods 

### 2.1. Sample Preparation

C-coated and C/B_4_C-coated SiC fibers about 100 μm in diameter (provided by AECC Beijing Institute of Aeronautical Materials, Beijing, China) were prepared by CVD. MCF method was employed to fabricate Ti_2_AlNb coatings onto tungsten-cored SiC monofilament as precursor wires in a facing-targets magnetron-sputtering system, with the Ti_2_AlNb alloy (nominal composition in at.% of Ti-22Al-25Nb) utilized as targets and the coated SiC fibers used as substrates. Next, these precursor wires were put into a Ti_2_AlNb alloy canister hermetically, which was then sealed using electron beam welding. Subsequently, the SiC_f_/Ti_2_AlNb was manufactured by consolidating those packed precursor wires through HIP under the soaking temperature, pressure, holding-time conditions of 970 °C, 150 MPa, 120 min, respectively, and then furnace cooled to the room temperature. This group of HIP process parameters is based on previous research [27]. With this process employed, the composite material can be completely closed, the diffusion barrier coating remains unfailing, and the RL will not be too intense. Clearly, after HIP, two composites evolving from C-coated and C/B_4_C-coated SiC fibers were named as SiC_f_/C/Ti_2_AlNb and SiC_f_/C/B_4_C/Ti_2_AlNb, respectively.

### 2.2. Characterization

**Figure 2:**
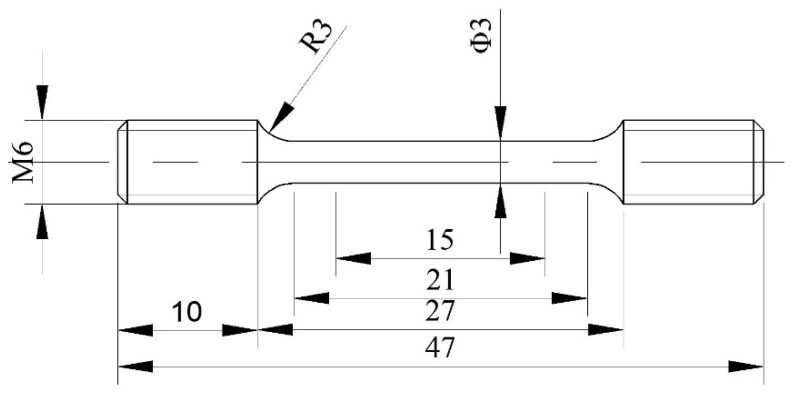
Tensile specimens of SiC_f_/Ti_2_AlNb.

The sectional morphology and chemical binding state of C and C/B_4_C coatings on SiC fibers were analyzed respectively by scanning electron microscope (SEM, FEI nano 450, Hillsboro, OR, USA), as well as ESCALAB-250 X-ray photoelectron spectrum (XPS, Waltham, MA, USA) using Al Ka radiation as the X-ray source with an energy of 1 keV. The consolidated composite specimens designated for microstructural observation by SEM were cut along the cross-section, polished, and etched in a 10% HF + 30% HNO_3_ solution. Specimens for transmission electron microscope (TEM) observation were cut from the composites in two directions, perpendicular to the cross-section and in the radial direction of the fiber, by FEI Quanta 200 FEG focused ion beam milling (FIB, Hillsboro, OR, USA) [28]. The interfacial reaction products were investigated by field emission JEOL 2010F TEM (Tokyo, Japan,) and the chemical compositions of the sub-layer in interfacial zones were further examined by energy-dispersive X-ray spectrometer (EDS) equipped in TEM. In order to study the kinetics of interfacial reaction, the composite samples were heat treated in vacuum at 800, 850, 900, and 950 °C for 36, 64, 100, 144, and 196 h, respectively. The total thickness of RL for these heat treatment samples was measured by SEM. Nanoindenter (Nano Test Xtreme, Micro Materials, Wrexham, UK) was employed to evaluate the interfacial debonding strength via fiber push-out test with the sample slices with 0.35 ± 0.01 mm thickness [29]. Two tensile samples were prepared for each HIP consolidated specimen with the gauge dimensions of Φ3 × M6, as shown in Figure 2, and the composite material inside the tensile specimen was ~2.8 mm in diameter. The room temperature tensile tests were performed at an extension rate of 1 mm/min, using the Inspekt Table 100 kN model universal testing machine. The fractographies were observed by SEM and laser scanning confocal microscope (LSCM, KEYENCE VK-100, Osaka, Japan).

## 3. Results and Discussion

### 3.1. Microstructure of C and C/B_4_C Coatings

**Figure 3:**
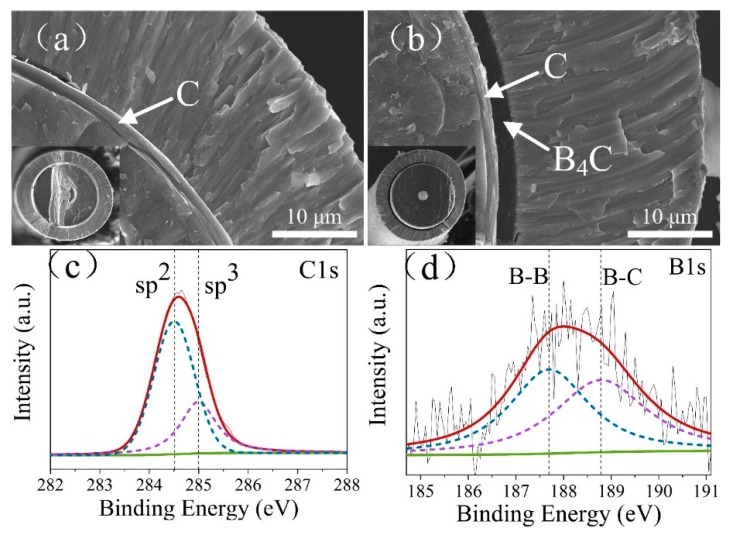
Cross-sectional SEM micrographs of SiC_f_/Ti_2_AlNb precursor wires for (**a**) SiC_f_/C/Ti_2_AlNb and (**b**) SiC_f_/C/B_4_C/Ti_2_AlNb; and typical XPS patterns for (**c**) C coating and (**d**) B_4_C coating.

**Figure 4:**
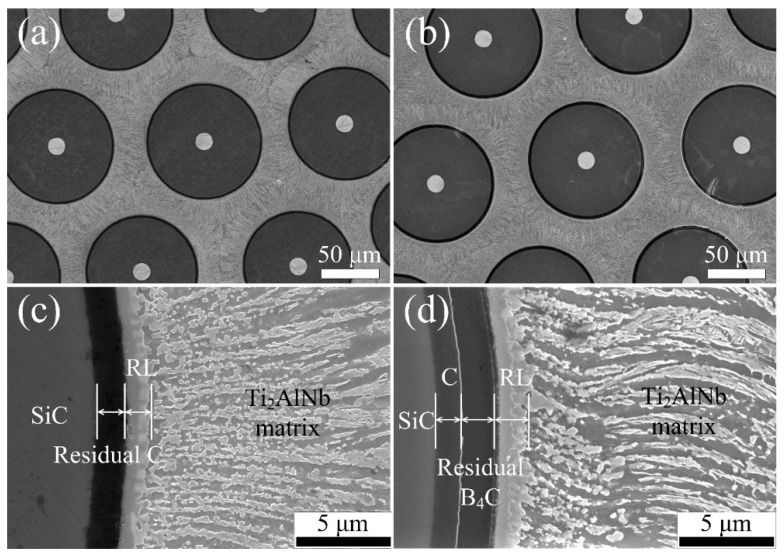
Microstructure of SiC_f_/Ti_2_AlNb observed by SEM for (**a,c**) SiC_f_/C/Ti_2_AlNb and (**b,d**) SiC_f_/C/B_4_C/Ti_2_AlNb.

**Figure 5:**
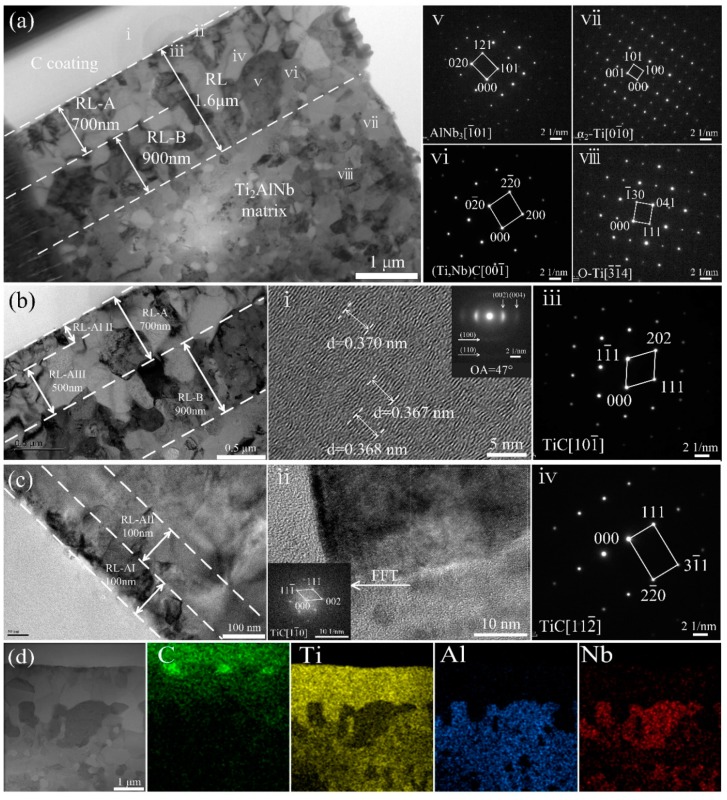
(**a–c**) TEM bright field images and high magnification images of interfacial reaction products for SiC_f_/C/Ti_2_AlNb; (**d**) STEM image and EDS mappings of the interfacial zones for SiC_f_/C/Ti_2_AlNb; (**i**–**viii**) shows the HRTEM images of C coating and SAED patterns of interfacial reaction products for SiC_f_/C/Ti_2_AlNb marked in (a).

As expected, C coating and C/B_4_C duplex coating were successfully fabricated on SiC fibers by CVD. The results of SEM (Figure 3a,b) reveal that the uniform-thickness C single coating and the C/B_4_C duplex coating can be clearly identified and were located between SiC fibers and Ti_2_AlNb matrix coatings. It is noted that the thickness of C single coating was ~1.5 μm, which was almost the same as the C layer-thickness in the C/B_4_C duplex coating, and the total thickness of the C/B_4_C duplex coating approached ~3.5 μm. The ~20 μm thickness Ti_2_AlNb coatings on both barrier coatings are clearly visible, which were deposited by PVD process and would evolve into the matrix during consolidation. The morphologies of the two composites after consolidation are shown in Figure 4. XPS was further used to investigate the chemical binding of the C and B_4_C coatings. For C coating, the XPS C 1s spectrum is presented in Figure 3c, which could be deconvoluted into two peaks located at ~284.5 and ~285.0 eV, corresponding to sp^2^-hybridized and sp^3^-hybridized carbon, respectively. As suggested by the following high-resolution TEM (HRTEM) in Figure 5ⅰ, the C coating was a typical pyrolytic carbon exhibiting a turbostratic carbon structure in nature. This structure consists of small basic structural units (BSUs) mainly comprising nanoscale few-layer graphene planes. Thus, the sp^2^-hybridized carbon should come from graphene planes in BSUs of the C coating [16]. In contrast, the sp^3^ peak should be assigned to defects in graphene planes or the amorphous region in pyrolytic carbon due to its nature of high-density defects [30,31]. The integral area ratio of sp^2^ and sp^2^ + sp^3^ was calculated to be 76.0%, which suggested that the C coating exists mainly in the form of sp^2^-hybridized graphene planes in BSUs and still possess high-density defects in graphene planes. For B_4_C coating, the XPS B 1s spectrum is shown in Figure 3d, and the deconvolution revealed the existence of two boron chemical states at the binding energies of 187.7 and 188.8 eV, corresponding to the B–B and B–C bonds, respectively [32]. This suggests that the B_4_C coating still possesses a large amount of B–B bonds.

**Figure 6:**
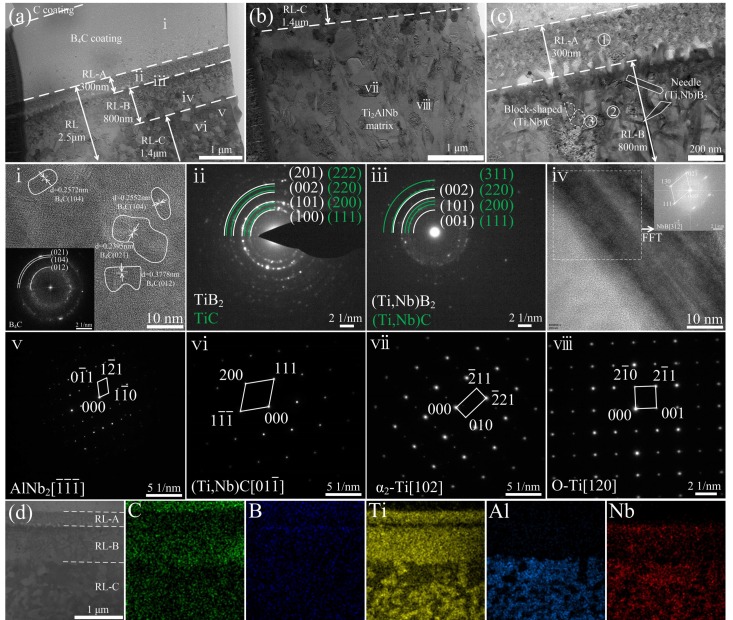
(**a–c**) TEM bright field images and high magnification images of interfacial reaction products for SiC_f_/C/B_4_C/Ti_2_AlNb; (**d**) STEM image and EDS mappings of the interfacial zones for SiC_f_/C/B_4_C/Ti_2_AlNb; (**i**–**viii**) shows the HRTEM images of B_4_C coating and SAED patterns of interfacial reaction products for SiC_f_/C/B_4_C/Ti_2_AlNb marked in (a) and (b).

HRTEM and selected-area electron diffraction (SAED) were further used to analyze the microstructure of C and B_4_C coatings, which are shown in Figure 5ⅰ and Figure 6ⅰ, respectively. It reveals that C coatings exhibited a typical turbostratic carbon structure with a certain degree of tortuosity in lattice fringes, in which BSUs contained seven to eight layers (d space: 0.368 nm) as marked in the HRTEM. According to the model proposed by Reznik et al., the SAED patterns further suggested the formation of high-texture carbon by the appearance of orientation angle (OA) of 47° [33,34]. In contrast, the B_4_C coating exhibited a typical polycrystalline structure with the random B_4_C nano-particles (~10 nm in average grain size) surrounded by a small amount of amorphous phases, as revealed by the HRTEM images and corresponding SAED in Figure 6ⅰ.

### 3.2. Interfacial Reactions in the SiC_f_/C/Ti_2_AlNb and SiC_f_/C/B_4_C/Ti_2_AlNb

Figure 4a,b show the cross-sectional overview of the SiC_f_/C/Ti_2_AlNb and SiC_f_/C/B_4_C/Ti_2_AlNb after consolidating at the same HIP condition. The fibers in both composites were uniformly arranged in a hexagonal distribution. Apparently, the interfacial reaction layers could be found between the residual barrier layer and the Ti_2_AlNb matrix in both composites, which might mainly come from the interdiffusion of elements between the adjacent layers during high-temperature HIP consolidation [2]. In the SiC_f_/C/Ti_2_AlNb, the RL was compact with no cracks or holes, and its thickness was evaluated to be ~1.6 μm. As C coating was utilized as the barrier layer of SiC_f_/Ti, the RL mainly arose from the continuous diffusion of C atoms from C coating into the adjacent Ti matrix and was essentially a TiC-based RL [16]. Near the RL, the Ti_2_AlNb matrix showed in a typical block or strip morphology. In the SiC_f_/C/B_4_C/Ti_2_AlNb, a thicker RL of ~2.5 μm in thickness formed between the B_4_C and Ti_2_AlNb matrix, as compared with that in the SiC_f_/C/Ti_2_AlNb. Compared to the SiC_f_/C/Ti_2_AlNb, the SiC_f_/C/B_4_C/Ti_2_AlNb allowed the boron atoms with a smaller size to easily diffuse in the gaps and the grain boundaries at a higher rate, consequently forming a thicker interfacial reaction layer. Clearly, besides the thickness of RL, the reaction products and their distribution in the RL can strongly impact on interfacial properties. In order to clarify the products distribution and the diffusion barriers-dominated interfacial reaction, the interfacial reaction zones were further observed by TEM

TEM bright field images of the interfacial reaction zones and the Ti_2_AlNb matrix in the SiC_f_/C/Ti_2_AlNb are shown in Figure 5. EDS analyses on concerned points are shown in Table 2. Based on the reaction products, the RL could be divided into two sub-layers, namely, RL-A near C coating and RL-B adjacent to the Ti_2_AlNb matrix. The RL-A was composed of TiC_x_, while the RL-B consisted of (Ti,Nb)C+AlNb_3_, as identified by the HRTEM and the SAED patterns in Figure 6ⅱ–ⅵ. Enlarged images of the RL-A are also displayed in Figure 5b,c. On basis of the size of TiC_x_ grains, the RL-A could be further divided into RL-AI (fine-grained TiC_x_ layer adjacent to C coating), RL-AII (transition layer of TiC_x_ in the middle of RL-A zone), and RL-AIII (coarse-grained TiC_x_ layer next to the RL-B). For SiC_f_/C/Ti_2_AlNb, the total thickness of RL-A was evaluated to be ~0.7 μm, consisting of ~100 nm RL-AI, ~100 nm RL-AII, and ~0.5 μm RL-AIII. Meanwhile, the grain size considerably increased from less than 100 nm for RL-AI to ~200 nm for RL-AII and ~500 nm for RL-AIII The gradual increase in TiC_x_ grain size along RL-A could be explained by the fact that C atoms continuously diffuse from the C coating toward the matrix and the matrix grains grow at the same time during HIP. A description for the process of RL formation is available in our previous paper [28]. Unlike the RL-A that was only composed of TiC_x_, the RL-B mainly comprised (Ti,Nb)C and AlNb_3_, as confirmed by SAED and EDS in Figure 5ⅴ,vi. The scanning TEM (STEM) images and EDS mapping analyses of elements were obtained to further visualize the elemental distribution of the interfacial zones of the SiC_f_/C/Ti_2_AlNb, as shown in Figure 5d. The metallic elements in the matrix were almost completely blocked and located outside the C coating. By contrast, C atoms diffused from the C coating toward the matrix, which dominated the formation of interfacial reaction zones with C content gradient. Obviously, the reaction products in RL-A were all TiC. Based on the above observations, the sequence of reaction products in SiC_f_/C/Ti_2_AlNb was TiC and (Ti,Nb)C+AlNb_3_. This can be discussed as follows. Compared with the FFF foils commonly used by many researchers, the PVD Ti_2_AlNb was composed of lots of nanocrystalline grains, which could provide a large amount of channels for elemental diffusion. Accordingly, the interfacial reaction was supposed to be a reaction- and diffusion-controlled process. Table 3 lists the equations for describing the possible interfacial reactions occurring in the SiC_f_/C/Ti_2_AlNb composites and the corresponding *ΔrG* values [35,36]. Reaction 5 has a more negative value of *ΔrG* than reaction 10. In addition, Ti atoms have a higher diffusion rate than Nb atoms to the C coating during the reaction [37]. They resulted in the formation of TiC in the initial stage of the reaction. At the same time, the remaining high concentration of Al and Nb elements in the Ti-depletion matrix formed AlNb_3_. During the reaction, the elemental diffusion occurred simultaneously with the grain growth. When the near-stoichiometric TiC formed and thus became a C-diffusion barrier, the interfacial reaction that followed would transit from the reaction-controlled to the diffusion-controlled. Since the C source was absolutely sufficient, only a small amount of C passed through the RL-A and was located at its front end, and reacted with the Ti and Nb in the matrix to form (Ti,Nb)C. In this process, the formation of the relatively-stable AlNb_3_ phase and (Ti,Nb)C resulted in an Al-enriched zone, in which the reaction with C did not occur, thereby increasing the transition temperature of B_2_ phase in Ti_2_AlNb matrix. Near RL-B, the Ti_2_AlNb matrix mainly consisted of α_2_ and O phases, as identified by SAED.

TEM bright field images and EDS analyses of the interfacial reaction zones in the SiC_f_/C/B_4_C/Ti_2_AlNb are shown in Figure 6a,b and Table 4, respectively. The RLs (~2.5 μm in total thickness) could be divided into three sublayers based on the reaction products, namely RL-A, RL-B, and RL-C. Enlarged images of the RL-A and the RL-B are also displayed in Figure 6c. The RL-A, adjacent to the B_4_C coating, was composed of fine-grained TiB_2_ and TiC with the grain size of ~20 nm, as revealed by the SAED patterns in Figure 6ⅱ. This means that only Ti reacted with B and C in the RL-A. Across the RL-A, both Ti and Nb reacted with B and C, forming the RL-B. Thus, the RL-B consisted mainly of the block-shaped (Ti,Nb)C and the needle-shaped (Ti,Nb)B_2_ with the elongated grains, as indicated by SAED and EDS in Figure 6ⅲ,ⅳ and Table 2. Unlike the carbide products with equiaxed feature, the boride products existed in needle shape when their composition deviated from the stoichiometry [15]. In the RL-C outside the RL-B, only the C atom diffused across RL-B, whereas the B atom diffusion terminated in RL-B, forming the coarse-grained (Ti,Nb)C with the grain size of ~100 nm, together with AlNb_2_, as revealed by SAED and EDS in Figure 6ⅴ,ⅵ. Figure 6d shows the STEM images and EDS elemental maps of the interfacial zones in the SiC_f_/C/B_4_C/Ti_2_AlNb for further visualizing the elemental distribution. All reactions occurred outside the B_4_C coating, and the diffusion of B and C atoms dispersedly distributed throughout the reaction zone and dominated the reactants. The Ti, Nb, and Al elements started to appear in RL-A, RL-B, and RL-C in turn due to their different reactivity. Additionally, the clear boundaries of B_4_C coating and each sublayer of interfacial reaction zones can be distinguished in the SiC_f_/C/B_4_C/Ti_2_AlNb. The sequence of reaction products in the SiC_f_/C/B_4_C/Ti_2_AlNb was TiB_2_+TiC, (Ti,Nb)B_2_+(Ti,Nb)C+NbB, and (Ti,Nb)C+AlNb_2_, and will be discussed in detail as follows.

**Figure 7:**
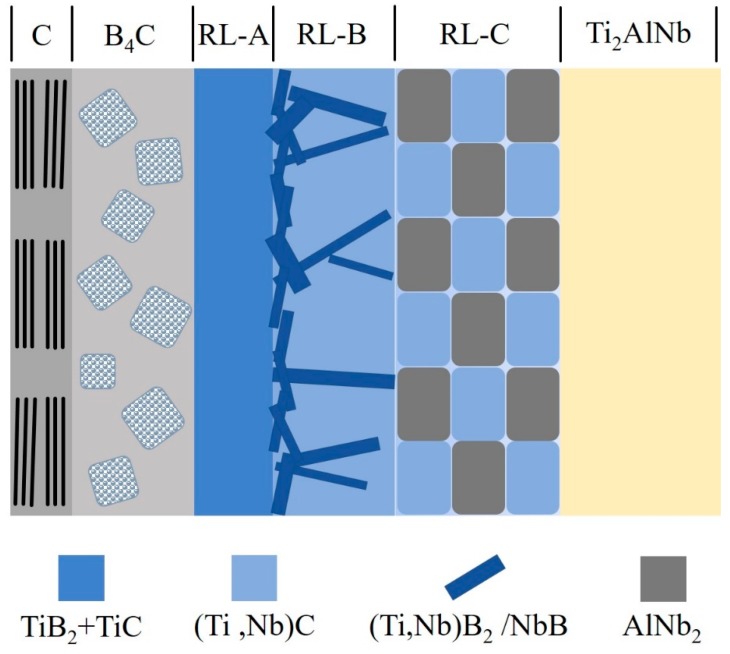
Schematic of interfacial reaction products in SiC_f_/C/B_4_C/Ti_2_AlNb composite.

The initial stage of the interfacial reaction was a reaction-controlled process. The reactions (1), (2), (5), and (3), as listed in Table 3, took place preferentially because of the high diffusion rate of Ti and the high absolute values of *ΔrG*, and resulted in the production of TiB_2_ and TiC near the B-rich B_4_C coating in the RL-A. High concentration of B-rich B_4_C contributed to the production with high nucleation rate that hindered the grain growth of TiB_2_ and TiC, and lots of fine grains provided terrible crystalline-boundary diffusion paths for B and C atoms in the RL-A. As the reaction and the diffusion of the elements proceeded synchronously, Nb atoms began to participate during the reaction. A large amount of near-stoichiometric boride and (Ti,Nb)C (Ti-rich) in needle and block shape, respectively, were formed in the RL-B. These products in RL-B could be regarded as the diffusion barrier layer, which led to the interfacial reaction entering the diffusion-controlled dominated stage. After the formation of previous products, the remnant highly-concentrated elements of Nb and Al in the Ti-depletion matrix formed AlNb_2_ in the RL-C. Compared with the AlNb_3_ formation in the RL-B of SiC_f_/C/Ti_2_AlNb, the AlNb_2_ formation could be attributed to a certain amount of participated Nb atoms during the preformation of the borides. Simultaneously, a small amount of carbon released by the reactions diffused into the matrix and formed a bit of (Ti,Nb)C. Due to the equilibrium of reaction process, a large amount of Al atoms were not involved in the reactions, leading to the Al-enriched matrix. Consequently, the main constituent phases of this matrix adjacent to the RL-C were α_2_-Ti and O-Ti. The sequence of reaction products in the SiC_f_/C/B_4_C/Ti_2_AlNb is accordingly summarized in the schematic diagram, as shown in Figure 7.

In summary, the comprehensive SEM and TEM observations make it clear that in both the SiC_f_/C/Ti_2_AlNb and the SiC_f_/C/B_4_C/Ti_2_AlNb composites, the RL zones were formed via a two-stage process: the reaction-controlled and the diffusion-controlled. The according reaction products were identified as the different-sized TiC and the coarse-grained (Ti,Nb)C+AlNb_3_ for the SiC_f_/C/Ti_2_AlNb, and the fine-grained TiB_2_+TiC, the needle-shaped (Ti,Nb)B_2_/NbB+(Ti,Nb)C and the coarse-grained (Ti,Nb)C+AlNb_2_ for the SiC_f_/C/B_4_C/Ti_2_AlNb. Compared with the C single coating, owing to the B_4_C acting as the reaction front in the C/B_4_C duplex coating, the needle-shaped boride products facilitated elements diffusion and; thereby, formed a thicker RL.

### 3.3. Reaction Kinetics of SiC_f_/C/Ti_2_AlNb and SiC_f_/C/B_4_C/Ti_2_AlNb

**Figure 8:**
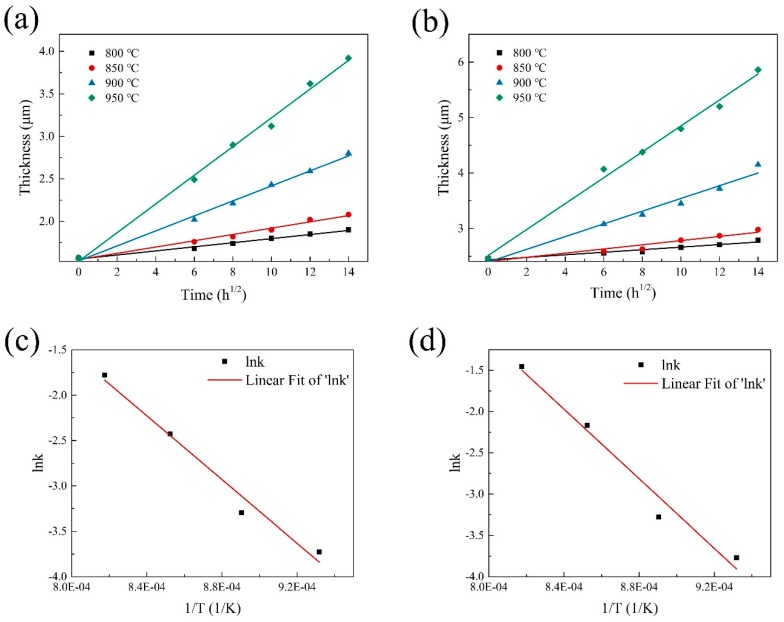
The total thickness of the reaction zone at various temperatures as a function of the square root of time for (**a**) SiC_f_/C/Ti_2_AlNb and (**b**) SiC_f_/C/B_4_C/Ti_2_AlNb composites; the logarithm of the reaction rates vs. reciprocal of the heat treatment temperature for (**c**) SiC_f_/C/Ti_2_AlNb and (**d**) SiC_f_/C/B_4_C/Ti_2_AlNb composites.

The interfacial reaction kinetics of SiC_f_/Ti is normally an indispensable and important basis for evaluating the thermal stability of SiC_f_/Ti and understanding the interfacial reaction mechanism of SiC_f_/Ti. The thickness of interfacial reaction zone develops progressively with the thermal exposure temperature and the increase of time. In detail, the thickness of RL is linearly related to the square of heat treatment time for the interfacial reaction zone in which the diffusion-controlled process dominates [38]. The total thickness of RL plotted as a function of the square root of reaction times at various heat treatment temperatures for the SiC_f_/C/Ti_2_AlNb and the SiC_f_/C/B_4_C/Ti_2_AlNb composites was estimated, respectively, and is shown in Figure 8a,b.

The reaction kinetics can be described by the following equations [39]:

*x* = *kt*^1/2^ + *b*_0_, (1)

*k* = *k*_0_*exp*(−*Q*/2*RT*) (2)

where *x* is the thickness of the RL, *t* is time, *k* is the reaction rate constant, *b_0_* is the original thickness of the RL, *R* is the gas constant, *Q* is the activation energy, *k_0_* is the frequency factor, and *T* is the temperature. The reaction rate k was determined from the slope of the curves for fitting the thickness of RL at different times. The logarithm of *k* vs. the reciprocal of *T*, according to Equation (2), was plotted and is shown in Figure 8c,d. The *Q* and *k_0_* of the interfacial chemical reactions were subsequently calculated.

The activation energy *Q* and the rate constant *k_0_* for the SiC_f_/C/Ti_2_AlNb and SiC_f_/C/B_4_C/Ti_2_AlNb composites were 292 kJ/mol, 0.457 cm/s^1/2^ and 350 kJ/mol, 11.4 cm/s^1/2^, respectively. Both the *Q* and the *k_0_* can normally affect the growth of the RL in SiC_f_/Ti. The *Q* of SiC_f_/C/B_4_C/Ti_2_AlNb was larger than that of SiC_f_/C/Ti_2_AlNb, indicating that the former would be more difficult to react in the early reaction-controlled stage. However, since the pyrolytic C coating with stronger texture could hinder the outward diffusion of C atoms and the needle-shaped borides could facilitate the elemental diffusion, the *k_0_* of SiC_f_/C/B_4_C/Ti_2_AlNb was much higher than that of SiC_f_/C/Ti_2_AlNb, which thereby resulted in a thicker RL of the former under the same consolidating conditions. Therefore, the growth rate of the RL in SiC_f_/Ti_2_AlNb is dependent on the interaction between the *Q* and the *k_0_*.

### 3.4. Interfacial Strength and Tensile Strength

**Figure 9:**
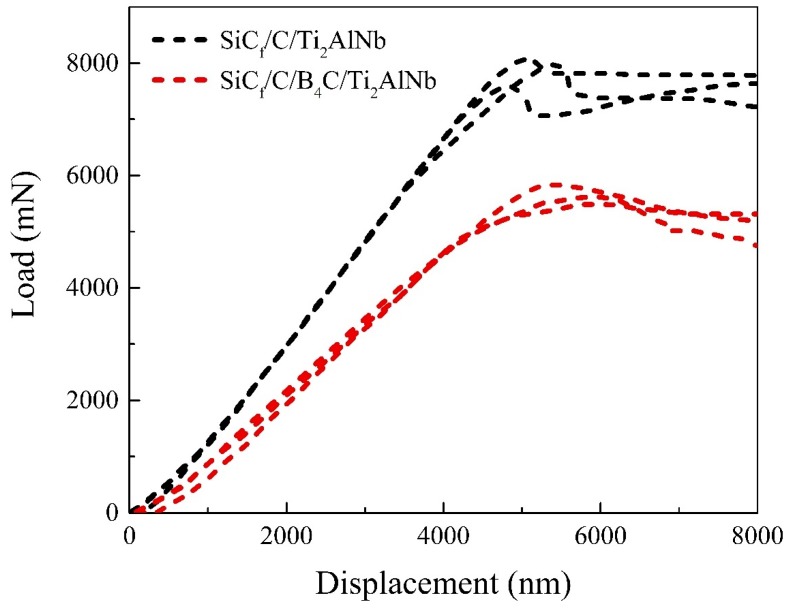
Typical fiber push-out load–displacement curves for SiC_f_/C/Ti_2_AlNb and SiC_f_/C/B_4_C/Ti_2_AlNb.

**Figure 10:**
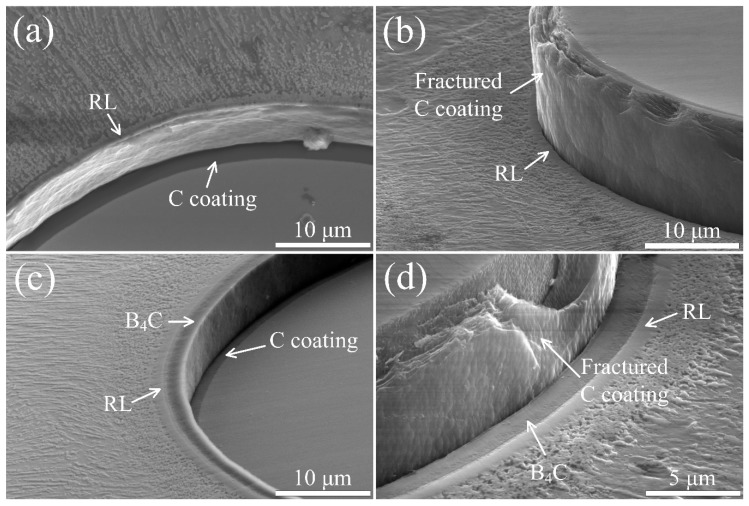
Appearance of the push-out fibers at the top and bottom of the test specimen for (**a,b**) SiC_f_/C/Ti_2_AlNb and (**c,d**) SiC_f_/C/B_4_C/Ti_2_AlNb.

The effect of RL thickness and reaction products on the SiC_f_/Ti composites were further explored by mechanical properties tests. It has been widely accepted that the interface strength of SiC_f_/Ti is a crucial parameter governing the failure modes of composites subjected to an external load, and thereby plays an important role in controlling the overall mechanical properties. Thus, push-out experiments were carried out to evaluate the interfacial properties of SiC_f_/C/Ti_2_AlNb and SiC_f_/C/B_4_C/Ti_2_AlNb composites. The load vs. displacement curve was determined and is shown in Figure 9. The interphase shear strength or interface strength, *τ_d_*, at the load level, *P_d_*, corresponding to the initial debonding, can be calculated based on the following formula [40]:

*τ_d_* = *P_d_*/*πd_f_h* (3)

where *d_f_* is the diameter of SiC fiber and *h* is the thickness of composite. The average values of *P_d_* for SiC_f_/C/Ti_2_AlNb and SiC_f_/C/B_4_C/Ti_2_AlNb were measured to be ~7.9 and ~5.6 N, respectively. By substituting *P_d_*, the calculated values of *τ_d_* were ~72 MPa for SiC_f_/C/Ti_2_AlNb and ~51 MPa for SiC_f_/C/B_4_C/Ti_2_AlNb. Fibers push-out were verified through SEM observations of the front and the reverse side of each specimen, as shown in Figure 10. The interfacial debonding took place at the location between the C coating and the RL for the SiC_f_/C/Ti_2_AlNb, and at the location between the C coating and the B_4_C coating for the SiC_f_/C/B_4_C/Ti_2_AlNb. It is generally believed that, within a certain thickness range, the *τ_d_* of the composites can be determined by the thickness of interfacial zone including diffusion barriers and RL. An elevated thickness of the interfacial zone appropriately would result in a higher value of *τ_d_* for SiC_f_/C/Ti_2_AlNb [16,27,28]. However, the interfacial debonding that occurred between pyrolytic carbon and amorphous polycrystalline structure of B_4_C resulted in a poor interfacial bond strength. Therefore, the *τ_d_* for SiC_f_/C/B_4_C/Ti_2_AlNb was lower than that for SiC_f_/C/Ti_2_AlNb, even though the former had a thicker interfacial zone.

**Figure 11:**
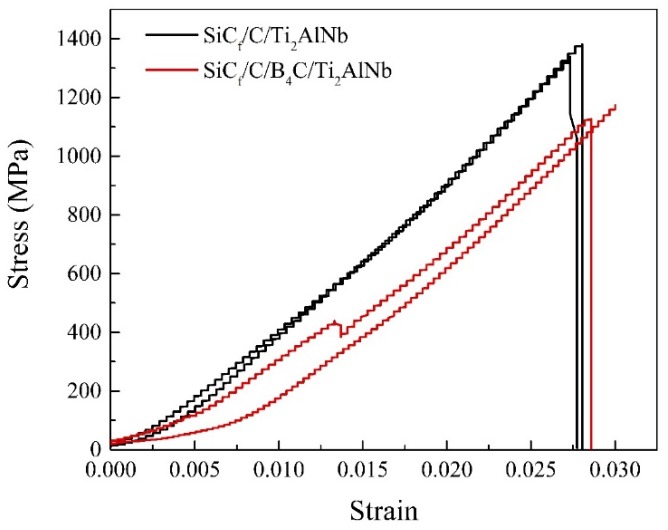
Typical tensile stress–strain curves of SiC_f_/C/Ti_2_AlNb and SiC_f_/C/B_4_C/Ti_2_AlNb.

**Figure 12:**
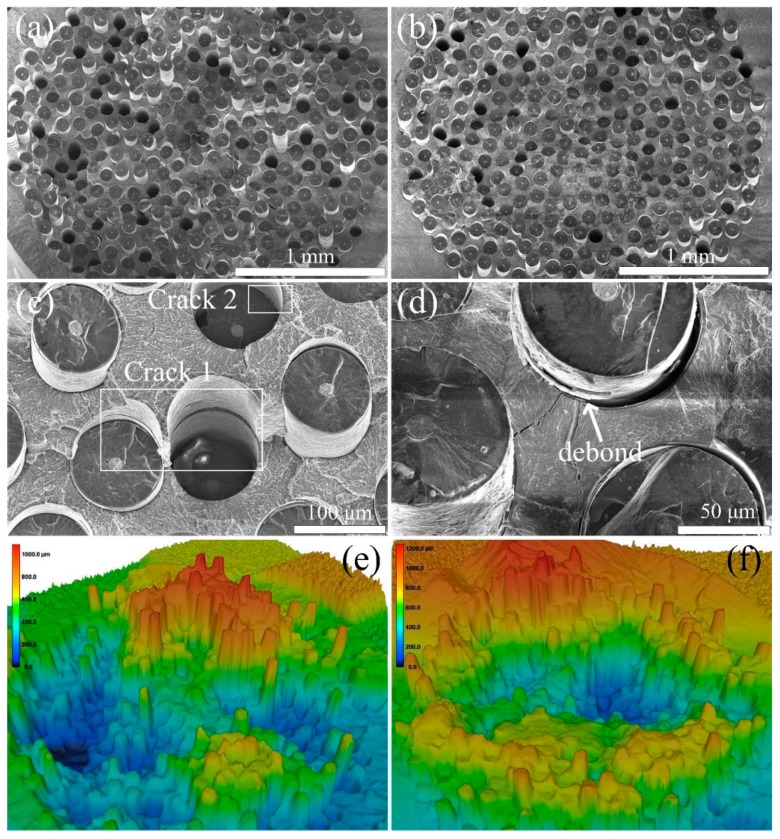
Low-magnification fractographs of (**a**) SiC_f_/C/Ti_2_AlNb and (**b**) SiC_f_/C/B_4_C/Ti_2_AlNb; high-magnification fractographs of (**c**) SiC_f_/C/Ti_2_AlNb and (**d**) SiC_f_/C/B_4_C/Ti_2_AlNb; fracture morphologies of (**e**) SiC_f_/C/Ti_2_AlNb and (**f**) SiC_f_/C/B_4_C/Ti_2_AlNb observed by LSCM.

We further carried out the room temperature tensile tests of the SiC_f_/C/Ti_2_AlNb and the SiC_f_/C/B_4_C/Ti_2_AlNb composites, with the tensile curves of SiC_f_/C/Ti_2_AlNb and SiC_f_/C/B_4_C/Ti_2_AlNb shown in Figure 11. The average tensile strength values of SiC_f_/C/Ti_2_AlNb and SiC_f_/C/B_4_C/Ti_2_AlNb were calculated to be ~1360 and ~1150 MPa, respectively. The tensile strength of SiC_f_/C/Ti_2_AlNb is ~200 MPa higher than that of SiC_f_/C/B_4_C/Ti_2_AlNb. This could be attributed to the difference in interfacial zone, although they had the same fibers and matrix alloy. Figure 12 shows the tensile fracture morphologies of SiC_f_/C/Ti_2_AlNb and SiC_f_/C/B_4_C/Ti_2_AlNb. Both two composites displayed an identical step-like fracture mode. This fracture behavior involved only the obvious push-out of short fibers; not the brush-like fracture caused by a too-low *τ_d_* nor the brittle facture induced by a too-high *τ_d_* due to lower interfacial debonding. However, in contrast to the fractograph of SiC_f_/C/B_4_C/Ti_2_AlNb, the fractograph of SiC_f_/C/Ti_2_AlNb exhibited more serious steps between the near regions of fiber and the surrounding matrix, corresponding to more obvious crack deflections. The difference in fracture characteristics are determined by the *τ_d_*. Compared with the SiC_f_/C/B_4_C/Ti_2_AlNb, more cracks usually initiated at the interfacial layers of the SiC_f_/C/Ti_2_AlNb, that has a higher *τ_d_* and better interface/matrix matching, as the process of continuous loading increased. When the crack passing through the brittle Ti_2_AlNb matrix propagated to the next interfacial layer, the crack easily changed the propagation direction and continued to extend along the plane of other cracks. This fracture behavior means that the crack would travel through a longer path and consume more fracture energy. Probably for this reason, the SiC_f_/C/Ti_2_AlNb had a higher tensile strength than the SiC_f_/C/B_4_C/Ti_2_AlNb, with more fiber bridges in the fracture morphology of SiC_f_/C/Ti_2_AlNb, as shown in Figure 12a. In a word, as the interfacial debonding totally dominated the tensile failure, the higher *τ_d_* would aid in improving the tensile strength.

## 4. Conclusions

Both C coating and C/B_4_C duplex coating were successfully fabricated onto SiC fibers by CVD and then consolidated into SiC_f_/C/Ti_2_AlNb and SiC_f_/C/B_4_C/Ti_2_AlNb after HIP. On the basis of microstructure observations and mechanical properties tests of two composites, we make the following conclusions:

(1) C-coated and C/B_4_C duplex-coated SiC fiber-reinforced Ti_2_AlNb composites were fabricated and the interfacial reaction products of both composites were identified. The reaction products sequence was the different-sized TiC and the coarse-grained (Ti,Nb)C+AlNb_3_ for the SiC_f_/C/Ti_2_AlNb; and the fine-grained TiB_2_+TiC, needle-shaped (Ti,Nb)B_2_/NbB +(Ti,Nb)C, and coarse-grained (Ti,Nb)C+AlNb_2_ for the SiC_f_/C/B_4_C/Ti_2_AlNb. When the interfacial reaction process of two composites was dominated by the diffusion-controlled, the SiC_f_/C/B_4_C/Ti_2_AlNb had a higher diffusion activation energy *Q* due to the facilitated elemental diffusion by the needle-shaped borides, making the frequency factor *k_0_* much larger than SiC_f_/C/Ti_2_AlNb, which resulted in a thicker RL in SiC_f_/C/B_4_C/Ti_2_AlNb under the same consolidating conditions.
(2) The SiC_f_/C/Ti_2_AlNb with a thinner RL exhibited a higher interface strength than SiC_f_/C/B_4_C/Ti_2_AlNb when the interfacial debonding occurred between pyrolytic carbon and the amorphous polycrystalline structure of B_4_C. The SiC_f_/C/Ti_2_AlNb with a higher interface strength exhibited a more serious step-like fracture than SiC_f_/C/B_4_C/Ti_2_AlNb and thus contributed to a higher tensile strength of the SiC_f_/C/Ti_2_AlNb.

## Figures and Tables

**Table 1:** Comparative properties of Titanium metal matrix composites (Ti MMCs) and superalloys.

Properties (Direction of Fibers)	Conventional Ti MMC	Ti Aluminide Ti MMC	Superalloys
Density, g/cm^3^	4.04	4.18	8.3
Stiffness, GPa	200	242	207
Max use temperature, ℃	538	760	1090
CTE, °C^−^^1^ × 10^-6^	8.91	9.18	13.0

**Table 2:** Chemical composition of interfacial reaction products in SiC_f_/C/Ti_2_AlNb composite (at.%).

Position	C	Ti	Al	Nb
a (ⅱ)	80.15	19.85	-	-
a (ⅳ)	76.54	23.46	-	-
a (ⅴ)	17.23	16.29	18.63	47.85
a (ⅵ)	59.75	33.85	0.40	6.00

**Table 3:** Equations of the interfacial reactions occurred in SiC_f_/C/Ti_2_AlNb and SiC_f_/C/B_4_C/Ti_2_AlNb composites and the corresponding *ΔrG* values at 970 °C.

No.	Interfacial Reaction Equation	*ΔrG*/kJ mol^−1^
1	2Ti + B_4_C = 2TiB_2_ + C	−230.5
2	Ti + 2B = TiB_2_	−258.7
3	Ti + B = TiB	−155.6
4	Ti + B_4_C = TiC + 4B	−111.9
5	Ti + C = TiC	−170.3
6	TiB_2_ + Ti = 2TiB	−52.4
7	2Nb + B_4_C = 2NbB_2_ + C	−133.7
8	Nb + 2B = NbB_2_	−162.9
9	Nb + B = NbB	−107.7
10	Nb + C = NbC	−134
11	4Al + 3C = Al_4_C_3_	−138

**Table 4:** Chemical composition of interfacial reaction products in SiC_f_/C/B_4_C/Ti_2_AlNb composite (at.%).

Position	B	C	Ti	Al	Nb
Spot 1 (RL-A)	65.14	14.10	20.76	-	-
Spot 2 (fine needle in RL-B)	62.25	9.50	13.88	0.23	14.13
Spot 3 (block in RL-B)	40.21	28.27	27.38	0.23	3.91

RL—reaction layer, The RLs could be divided into three sublayers based on the reaction products, namely RL-A, RL-B, and RL-C.
